# Assessment of lifestyle changes during coronavirus disease 2019 pandemic in Gondar town, Northwest Ethiopia

**DOI:** 10.1371/journal.pone.0264617

**Published:** 2022-03-18

**Authors:** Zemene Demelash Kifle, Alem Endeshaw Woldeyohanins, Biniyam Asmare, Birhanu Atanaw, Tigist Mesafint, Meaza Adugna

**Affiliations:** 1 Department of Pharmacology, School of Pharmacy, College of Medicine and Health Science, University of Gondar, Gondar, Ethiopia; 2 Department of Pharmaceutics and Social Pharmacy, School of Pharmacy, University of Gondar College of Medicine and Health Sciences, Gondar, Ethiopia; Debre Tabor University, ETHIOPIA

## Abstract

**Background:**

Coronavirus disease 2019 has had a global effect on people’s lifestyles. Many people have developed irregular eating patterns and become physically inactive, which leads to an aggravation of lifestyle-related diseases and unhealthier lifestyles; these, subsequently raise the severity of coronavirus disease 2019. This study aimed to assess lifestyle changes during coronavirus disease 2019 pandemic in Gondar town, North West, Ethiopia.

**Method:**

Community-based cross-sectional study design was conducted among households at Gondar town from June to August 2021. The study participants were selected by a systematic random sampling technique from proportionally allocated kebeles. Data were collected using face-to-face interview techniques and were entered and analyzed by using a statistical package for the social sciences version 24; *P*-values < 0.05 were considered as statistically significant.

**Result:**

Overall, 348 study participants were included in the study. Among those respondents, 52.3% (182) were female study participants and the mean age of the respondents was 30.95±14.4. In this study, there was a significant decrement in non-homemade food from 20.4% to 13.4% at (*P* = <0.001). Concerning water intake, 11.5% (40) of respondents consumed ≥8 cups/day before the coronavirus disease 2019 pandemic, and the percentage increased to 14.7% (51) during the coronavirus disease 2019 pandemic (p = 0.01). Of the participants, 46% participants were reported never engaging in any physical activity before the coronavirus pandemic, and the percentage decreased to 29.9% during the pandemic (*P* = 0.002). The respondents also exhibited increment tension in large from 4.9% to 22.7% before and during the coronavirus disease 2019 pandemic, respectively. Furthermore, about 6.3% of the study participants slept badly before the coronavirus disease 2019 pandemics and the effects of sleeping badly and restlessly increased to 25.9% during the coronavirus disease 2019 pandemic (*P* = <0.001).

**Conclusion:**

The current study demonstrates that there is a noticeable alteration in food consumption, food choices, regular mealtime, sleeping habits, mental exhaustion, and practice of physical activity.

## 1. Introduction

Coronavirus disease (COVID-19) is an infectious disease caused by severe acute respiratory syndrome coronavirus 2 (SARS-CoV-2) [1]. The novel COVID-19 pandemic has added various changes and challenges to human life worldwide, causing a significant impact on human social life, health, lifestyle, and economy [2,3]. The virus was first identified in December 2019, an outbreak of pneumonia caused by a novel coronavirus occurred in Wuhan, the capital of Central China, and has been declared a public health emergency of international concern by the World Health Organization since January 2020 [4,5]. Later on, it continues to spread across the world and affects about 200,000 people worldwide immediately after its emergence [5].

COVID-19 pandemic has changed lifestyles dramatically, with many people working from home and having little contact with people other than family members. These changes have possibly led to unhealthier lifestyles, altered rhythms of daily life, and less physical activity [6].

Covid-19 can alter, lifestyle behaviors, nutritional habits, and mental health [6,7]. Lifestyle disturbances include increased daily sitting time, changes in levels of physical activity, and altered sleep [6,8,9]. Also, mental health status is influenced by COVID-19 related restrictions such as social distancing and prolonged isolation, with increased depression, anxiety, and stress [7,10], which may also disrupt lifestyle behaviors. Moreover, the stress created by the pandemic condition, the occurrence of a threat perceived as novel, can also affect anxiety levels. Altogether, such changes, along with extended unstructured time, can affect human dietary behaviors and lead to weight gain during lockdown [8,9].

COVID-19 is not only a deadly disease outbreak but also affects the mental, social activity, eating, sleeping, and level of physical activity of the population [11–13]. And, now WHO declared the people to take a vaccine to reduce the transmission burden of the virus [14]. The emergence of COVID-19 reaching pandemic levels persuades huge distressing mental health symptoms and psychological impact in the people with anxiety being the most common as was revealed following SARS-CoV-2 and MERS-CoV [15,16]. Anxiety and hesitation along with restricted healthcare access and food insecurity could also impact people with obesity and eating disorders [17,18]. Several factors affect the extent of the psychological impact of outbreaks such as media misinformation, future unpredictability, quarantine, and unknown means of virus transmission [16,19]. Subsequently, such stressful events significantly worsen insomnia and disturbed sleep patterns, poor eating habits along with increased sedentary behaviors and decreased levels of physical activity [20,21]. Thus, this study aimed to assess lifestyle changes during COVID 19 pandemic in Gondar town, North West, Ethiopia.

## 2. Methods

### 2.1. Study design, period, and study area

A community-based cross-sectional study design was conducted from June to August 2021 in Gondar town. The study was conducted on individuals aged >18 years who live in Gondar town. The town in which the study was conducted is located 727km away from Addis Ababa, the capital city of Ethiopia, and 175km from Bahir Dar, the capital city of Amhara Regional State. Based on the Central Statistical Agency (CSA) report of Ethiopia, Gondar town has about 351, 675 total population [22]. Gondar town has 23 kebeles and the study was conducted on randomly selected 8 Kebeles.

### 2.2. Population and sampling procedure

All households in Gondar town were the source population, whereas households in the selected kebeles of Gondar town were the study population. The included study participants were all adults older than 18 years old who lived in Gondar town. However, participants with a previous diagnosis of sleep or psychiatric disorder, chronic systemic disease, hearing problem, and pregnancy were excluded.

The sample size determination was made using the single population proportion formula with the following assumptions: a proportion of 71% [23], a 95% confidence interval, a 5% margin of error, and 10% for non-response rate which gave a final sample size of 348. Then the total sample size, 348, was proportionally allocated among the eight selected kebeles based on the size of the households. From a total of 23 kebeles, eight kebeles were selected using a lottery method. The study participants were selected using a systematic random sampling technique from each household after getting a list of households from each kebele’s administration. Accordingly, the interval was determined by dividing the total households to the total sample sizes and the first household was selected through the lottery method among households within the first range of interval. If a selected household was not accessible, the next household was included. When two or more participants were identified in the same household, one participant was chosen using the lottery method. A family member aged 18 years and above was the respondent whenever the households were not available at the time of data collection.

### 2.3. Data collection tools and procedures

Data was collected using a validated interview-based questionnaire and different published articles were reviewed to prepare the data collection tool [24–30]. The questionnaire contains socio-demographic characteristics, eating habits, physical activity, stress and irritability, and sleeping habits of COVID-19. Before starting interviewing the questionnaires primarily being prepared in English then translated to local language (Amharic) for its consistency and understandability to the patients in which this research conducted. During translation, all the concerns and the local linguistic had been put into consideration and the local language (Amharic) was translated back to English for the appropriateness and conformability of analysis the finding of the research. Pretest was conducted among 5% of the participants from Metema town and modification had been considered according to its findings. The reliability of the questionnaire was checked with a Cronbach’s Alpha value of 0.768. After modification and amendment of the data was collected by face-to-face interview by two clinical pharmacy professionals after they obtain a one-day training on the tools and necessary care needed. The study participants were interviewed at the residence of the participants.

### 2.4. Operational definitions

#### Lifestyles

The way of living of human beings [31].

#### Lifestyle changes

Behavior modifications or habit changes that encourage positive life changes [32].

#### Eating

Ingestion of food or fluid [33].

#### Physical activity assessment

A revised version of the International Physical Activity Questionnaire Short Form was used to evaluate the frequency of physical activity before COVID-19 and during COVID-19 among respondents [34]. Respondents were asked to indicate “how many hours per day did they spend on the computer for work or study”, “how many days per week did they engage in moderate to vigorous physical activity”, “how many hours per day did they spend on screens for fun and entertainment”, and “how many days per week did they engage in household chores”.

#### Stress, irritability, and sleep assessment

The modified version of the Copenhagen Psychosocial Questionnaire with modifications was used to assess the stress and sleep pattern of the respondents before COVID-19 and during COVID-19 among respondents [35]. Concerning stress and irritability, respondents were asked to provide the frequency of experiencing emotional irritability, exhaustion, tension, and physical exhaustion. The same questions were asked pre-COVID-19 and during COVID-19. Regarding sleep, respondents were asked if they experienced sleep disturbances such as restlessly and sleeping badly; having difficulty to go to sleep; waking up several times and found it difficult to get back to sleep; waking up too early and not being able to get back to sleep; or none of the options. The questionnaire also comprised the following questions: “rating sleep quality”, “number of sleeping hours per night”, and “describing energy level during the day”. The repose options for describing energy level were energized; neutral; lazy. The repose options for rating sleep quality were very good; good; poor.

#### Perceived health state

A commonly used measure is the person’s rating of his or her own general health, as in the five-category classification, excellent, very good, good, fair, or poor. Used in the National Health Interview Survey and many other studies, this item has been shown to be predictive of morbidity, mortality, and future medical care use. Excellent health state-this category includes persons who rated their health as excellent or whose health was rated as excellent by someone knowledgeable about them. This is the most positive rating on the perceived health state. Very good-this category includes persons who rated their health as very good or whose health was rated as very good by someone knowledgeable about them. The rating very good comes between excellent and good. Good- this category includes persons who rated their health as good or whose health was rated as good by someone knowledgeable about them. This is the midpoint on the perceived health scale which is a five-point scale ranging from excellent to poor. Fair- this category includes persons who rated their health as fair or whose health was rated as fair by someone knowledgeable about them. The rating fair comes between good and poor. Poor- this category includes persons who rated their health as poor or whose health was rated as poor by someone knowledgeable about them. This is the most negative rating on the perceived health scale, a five-point scale ranging from excellent to poor [36,37].

### 2.5. Data processing and analysis

Before analysis of the data, the collected data was checked for its completeness, consistency, and validity. Then the data was cleaned and entered to EPI-info version 7.2.1 and transferred to Statistical Package for the Social Sciences (SPSS) version 24 statistical software for analysis. Analysis of the data concerned with the descriptive and analytical part. For analytical presentation frequency, cross-tabulation and texts were considered. For the analytical part, the significant changes were before and during the COVID-19 pandemic were tested by paired t-test. P-value < 0.05 at 95% confidence interval had been considered as statistically significant. Model goodness-of-fit was determined by, if P-value <α- is possible to (reject the null hypothesis) and there is a significant difference between the paired independent variables.

### 2.6. Data quality control

The quality of data was ensured by doing the questionnaire pre-tested on 5% of the total sample size at Metema town that is assumed to have similar characteristics to the targeted population. Based on the feedback obtained from the pretest of the respondent’s interview, the necessary amendment was done and the questionnaire was assessed for its clarity; the completeness and evaluate the validity and content of the questionnaire and modified accordingly. Close supervision had been made by the supervisor during the data collection and appropriate feedback was provided. The training was provided to the data the 2 collectors for one day by the principal investigator and the training was focused on the objective, how to obtain consent, keeping the confidentiality of the information they gathered. The collected data was cheeked for its completeness every day before the following day of data collection by supervisors and the principal investigator and corrective measures were taken according to the finding during supervision.

### 2.7. Ethical consideration

Ethical clearance was obtained from the Ethical Review Committee of College of Medicine and Health Science, School of Pharmacy, the University of Gondar with a reference number of SOP/262/2021, and the study was also conducted following the Declaration of Helsinki. Informed written consent was obtained from the study participants at the beginning of filling the survey. The information collected from respondents was kept confidential and there were no personal identifiers in the questionnaire.

## 3. Results

### 3.1. Socio-demographic characteristics and other background information of the study participants

Overall, 348 study participants were included in the study and it produces a response rate of 100%. Among those respondents, 52.3% (182) were female study participants and the mean age of the respondents was 30.95±14.4, whereas more than one-third of the study subjects 34.5% (120) were found within the age group of 18–25 years. Of the total study participants, 71.8% (250) were orthodox in their religion, and about half of the study participants 48.9% (170) engaged with marriage, whereas 51.7% (180) had no children. Regarding educational status, 25% (87) of the study participants college/diploma by their educational status. Of all respondents, more than one-third 30.5% (106) of the study participants were full-time by their employment status. On the other way, 45.1% (157) of the study participants earn <2500 ETB monthly, whereas less than one-third 29.6% (102) of the study subjects maintain their weight. Similarly, 43.4% (151) of them perceive a very good state of health during the COVID-19 pandemic (**Table 1).**

**Table 1:** Socio-demographic characteristics and other background information of the respondents.

Variables	Frequency	Percentage (%)
**Age categories (years)**		
18–25	120	34.5
26–35	94	27.0
36–45	74	*21*.*3*
46–55	39	11.2
>55	21	6.0
**Gender**		
Female	182	52.3
Male	166	47.7
**Marital status**		
Married	170	48.9
Single	151	43.4
Divorced	10	2.9
Widowed	17	4.9
**Religion**		
Orthodox	250	71.8
Muslim	64	18.4
Protestant	34	9.8
Others	0	0
**Number of children**		
1–2	79	22.7
≥ 3	89	25.6
None	180	51.7
**Education level**		
Less than high school	34	9.8
College/Diploma	87	25
High school	63	18.1
Higher than bachelor’s degree	26	7.5
Bachelor’s degree	65	18.7
Illiterates	73	21
**Employment status**		
Part-time	9	2.6
Full-time	106	30.5
Self-employed	102	29.3
Student	67	19.3
Unemployed	45	12.9
Retired	19	5.5
**Amount of money you earn per month by any means**		
< 2500 birr	157	45.1
2500–5000 birr	103	29.6
>5000 birr	88	25.3
**Weight change during a pandemic**		
Gained weight	68	19.5
Maintained weight	103	29.6
Lost weight	69	19.8
Do not know	108	31.0
**Perceived health state during a pandemic**		
Excellent	94	27.0
Very good	151	43.4
Fair	25	7.2
Good	77	22.1
Poor	1	0.3

### 3.2. Sources of information

Of the total 348 study participants, less than one-third 29% (101) of them got health-related information from mass media like television, whereas 27.6% (96) of the study subjects got a source of food and nutrition-related information from television. Furthermore, healthcare professionals were selected as the second source of information for Health-related information 21.6% (75) and social media for nutrition-related information 15.5% (61). (**Table 2).**

**Table 2:** Sources of information of the study participants.

Source of Information	Health-related information, n%	Nutrition-Related Information, n%
Local and international health authorities	52 (14.9)	54 (15.5)
Social media	55 (15.8)	61 (17.5)
Healthcare professionals	75 (21.6)	48 (13.8)
Newspapers	14 (4)	43 (12.4)
Television	101 (29)	96 (27.6)
Friends and family	51 (14.7)	46 (13.2)

### 3.3. Eating habits

Concerning meal consumption of the respondents, there is a significant decrement of non-homemade food from 20.4% to 13.4% at (<0.001). However, there is an increment in the frequency of food consumption ≥5 meals by 2% (0.001) during the COVID-19 pandemic. Unfortunately, there is a significant decrement in skipping breakfast from 29.9% to 27.3% (<0.001) during the COVID-19 pandemic, but there is no change in skipping meals during COVID-19 (0.72). Concerning water intake, 11.5% (40) of respondents consumed ≥8 cups/day before the COVID-19 pandemic, and the percentage increased to 14.7% (51) during the COVID-19 pandemic (p = 0.01) **(Table 3)**.

**Table 3:** Eating habits pre- and during COVID-19 pandemic (n = 348).

Variables	Pre-COVID-19 n (%)	During-COVID-19 n (%)	p-Value (2-Sided)
Homemade	277 (79.6)	302 (86.8)	<0.001
None-homemade	71 (20.4)	46 (13.2)	<0.001
**Number of meals per day**			
1–2 meals	97 (27.9)	83 (23.9)	0.003
3–4 meals	241 (69.3)	248 (71.3)	0.56
≥5 meals	10 (2.9)	17 (4.9)	0.01
**Eating breakfast on** most days			
Yes	244 (70.1)	253 (72.7)	0.55
No	104 (29.9)	95 (27.3)	<0.001
**Skipping meals**			
Yes	164 (47.1)	159 (45.7)	0.72
No	184 (52.9)	187 (53.7)	0.63
**Reasons for skipping meals**			
Lack of appetite	26 (7.5)	26 (7.5)	1
Lack of time	37 (10.6)	35 (10.1)	0.9
To lose weight	30 (8.6)	23 (6.6)	0.75
Fasting	92 (26.4)	83 (23.9)	0.57
To reduced food intake	27 (7.8)	29 (8.3)	0.87
Amount of water consumed per day			
1–4 cups	202 (58)	186 (53.4)	0.032
5–7 cups	106 (30.5)	111 (31.9)	0.02
≥8 cups	40 (11.5)	51 (14.7)	0.01

### 3.4. Consumption of particular foods during COVID-19 pandemic

In this study, about 44.8% (156) of the respondents feed fruit 1–4 times/week, whereas 47.1% (164) of the study participants consume vegetables 1–4 times/week. Regarding milk and milk product consumption, 21.3% (74) of them feed milk and milk products once/day. Of the respondents, more than two-third 43.7% (152) respondents feed meat/chicken/fish 1–4 times/week, whereas more than one-third 37.4% (130) of the study subjects feed beard/rice/pasta 1–4 times/week. Regarding sweets/desserts, 35.3% (123) of the respondents consumed sweets and desserts at least once per day, whereas 35.9% (125) of the study subjects use sweet drinks (soft drinks, canned juice 1–4 times/week. Of the respondents, 31.6% (110) did not consume milk and milk products (**Table 4).**

**Table 4:** The frequency of consumption of particular foods during the COVID-19 pandemic (n = 348).

Food Items	Never n (%)	1–4 Times/Week n (%)	Once/Day n (%)	2–3 Times/Day n (%)	≥4 Times/Day n (%)
Fruits	76 (21.8)	156 (44.8)	84 (24.1)	13 (3.7)	19 (5.5)
Vegetables	38 (10.9)	164 (47.1)	88 (25.3)	42 (12.1)	16 (4.6)
Milk and milk products	110 (31.6)	108 (31.0)	74 (21.3)	42 (12.1)	14 (4.0)
Meat/fish/chicken	77 (22.1)	152 (43.7)	80 (23.0)	24 (6.9)	15 (4.3)
Bread/rice/pasta	30 (8.6)	112 (32.2)	128 (36.8)	48 (13.8)	30 (8.6)
Sweets/desserts	36 (10.3)	74 (21.3)	123 (35.3)	54 (15.5)	61 (17.5)
Coffee/tea	36 (10.3)	74 (21.3)	123 (35.3)	54 (15.5)	61 (17.5)
Sweetened drinks	121 (34.8)	125 (35.9)	51 (14.7)	41 (11.8)	10 (2.9)

### 3.5. Shopping

Concerning shopping, 62.1% (216) of the respondents were prepared the list of grocery shopping before the coronavirus pandemic. However, during the COVID-19 pandemic preparation of grocery shopping lists increased to 62.6% (218). Of the overall study participants, more than one-third 39.4% (137) participants started stocking up on food before the COVID-19 pandemic and increased start stocking up on food during the COVID-19 pandemic to 48% (167). Findings of the current study also revealed that the order of groceries online (delivered to house) before coronavirus pandemic was 23.9% (83), whereas the order of groceries online (delivered to house) during coronavirus pandemic also increased to 24.7% (86) during COVID -19 pandemic. On the other hand, 48.3% (168) of the study subjects check food labels before purchasing before coronavirus pandemic and increased to 57.2% (199) checking food labels before purchasing during coronavirus pandemic. Of the study subjects, more than two-third 77.2% (269) of the respondents cleaned and sanitize groceries before storage before the coronavirus pandemic, whereas the result increases to 83% (289) during the COVID-19 pandemic (**Table 5).**

**Table 5:** Shopping practices during COVID-19 pandemic (n = 348).

Variables	Frequency	Percentage (%)
**Prepare shopping list before a pandemic**		
Yes	216	62.1
No	132	37.9
**Prepare shopping list during a pandemic**		
Yes	218	62.6
No	130	37.4
**Start stocking up on foods before a pandemic**		
Yes	137	39.4
No	211	60.6
**Start stocking up on foods during a pandemic**		
Yes	167	48
No	181	52
**Online grocery shopping before a pandemic**		
Yes	83	23.9
No	265	76.1
**Online grocery shopping during a pandemic**		
Yes	86	24.7
No	262	75.3
**Checking food labels before a pandemic**		
Yes	168	48.3
No	180	51.7
**Checking food labels during a pandemic**		
Yes	199	57.2
No	149	42.8
**Clean and sanitize groceries before a pandemic**		
Yes	269	77.3
No	79	22.7
**Clean and sanitize groceries during a pandemic**		
Yes	289	83
No	59	17

### 3.6. Physical activity

In this study, about 46% of the respondents reported never engaging in any physical activity before the coronavirus pandemic, and the percentage decreased to 29.9% during the pandemic (p = 0.002). Moreover, subjects who perform a certain exercise before the COVID-19 pandemic in household chores showed a significant increment in everyday household exercise from 43.1% before the COVID-19 pandemic to 53.2% during the COVID-19 pandemic (<0.001). On the other hand, 33.6% of the study participants were spent 1–2 hours on the computer for work/ study daily before the coronavirus pandemic, whereas during the COVID-19 pandemic the report decreased to 23.3% (<0.01). However, the percentage of respondents spending 3–5 h/day on screen for TV and entertainment during the COVID-19 pandemic was increased from 26.4% (before COVID-19) to 33% (during COVID-19) pandemic (0.03) (**Table 6).**

**Table 6:** Daily activities pre-and during COVID-19 pandemic (n = 348).

Variables	Pre-COVID-19, n (%)	During COVID-19 n (%)	p-Value (2-Sided)
**Any Exercise**			
1–3 times/week	141 (40.5)	158 (45.4)	0.023
>3 times/week	47 (13.5)	86 (24.7)	0.023
Never	160 (46.0)	104 (29.9)	0.002
**Doing household chores**			
Never	73 (21)	38 (10.9)	<0.001
1–3 times/week	78 (22.4)	78 (22.4)	0.78
4–5 times/week	47 (13.5)	47 (13.5)	0.9
Everyday	150 (43.1)	185 (53.2)	<0.001
**Screen time for study or work**			
None	104 (29.9)	108 (31.0)	0.23
3–5 h/day	81 (23.3)	87 (25.0)	0.05
More than 5 h/day	46 (13.2)	72 (20.7)	0.04
1–2 h/day	117 (33.6)	81 (23.3)	<0.01
**Screen time for TV and entertainment**			
Less than 30 min	62 (17.8)	28 (8.1)	0.05
1–2 h/day	149 (42.8)	108 (31)	0.52
3–5 h/day	92 (26.4)	115 (33)	0.03
More than 5 h/day	45 (12.9)	97 (27.9)	0.04

### 3.7. Stress and irritability

**Fig 1:**
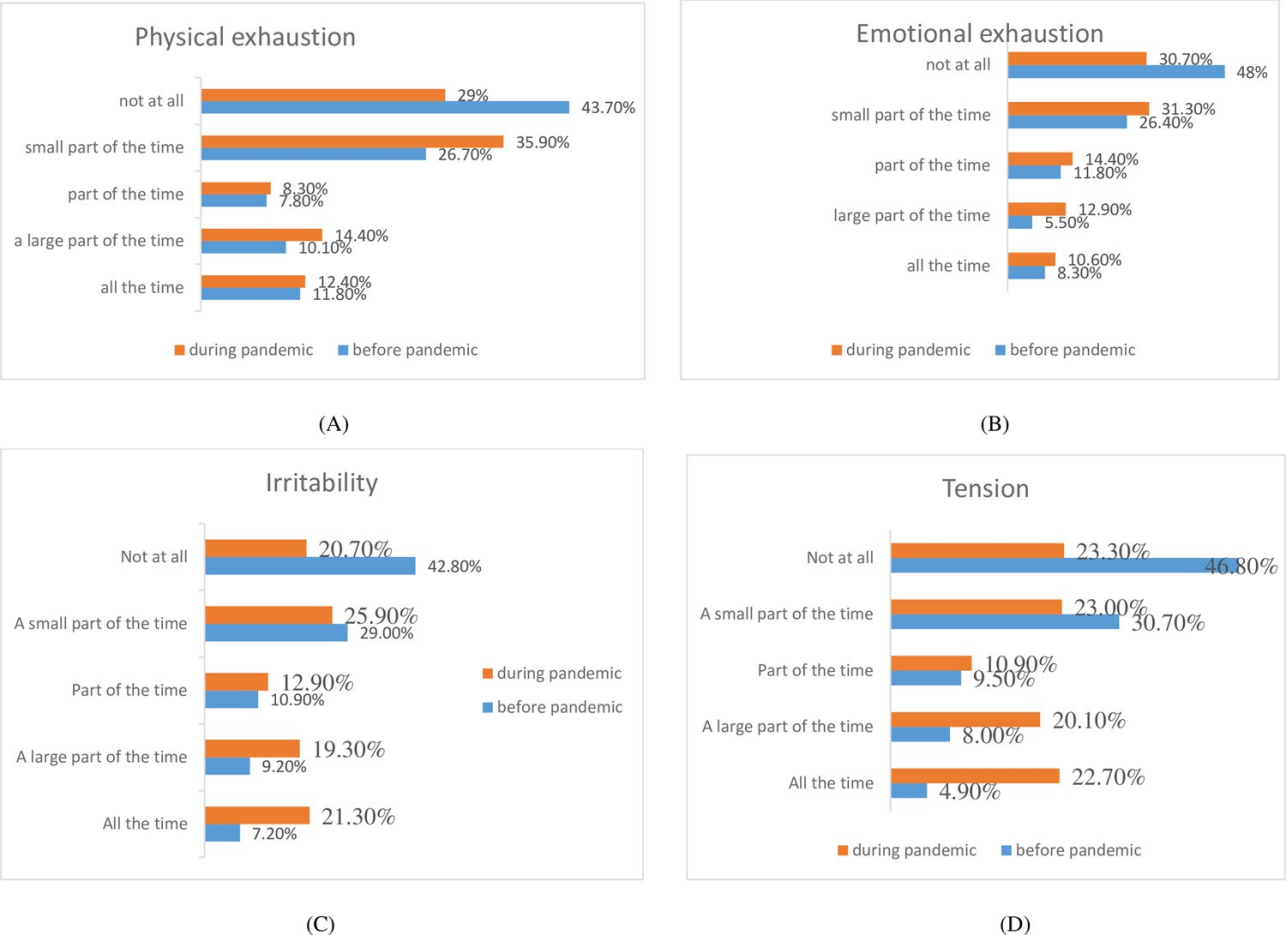
Stress and irritability pre-and during COVID-19 pandemic (A) Physical exhaustion; (B) motional exhaustion; (C) Irritability; (D) Tension.

Stress and irritabilities are a sign and symptoms of different physical and emotional disturbances. About 11.8% and 12.4% of the study participants were physically exhausted in all parts of the time before and during the COVID-19 pandemic, respectively. Of the participants, 8.3% and 10.6% participants were emotionally exhausted in all parts of the time before and during the COVID-19 pandemic, respectively. However, the level of irritability before the COVID -19 pandemic increased from 7.2% to 21.3% during the COCVID-19 pandemic. The respondents also exhibited increment of tension in large from 4.9% to 22.7% before and during the COVID-19 pandemic, respectively (**Fig 1).**

### 3.8. Sleep

Before the coronavirus pandemic, about 35.9% of the respondents have a night sleep <7 hrs, but the effects reduced to 35.1% during coronavirus pandemic (<0.001). Regarding sleeping quality, before the coronavirus pandemic, 43.7% and 8.3% have very good and poor sleep quality, respectively. However, the report of poor quality of sleep increased by 28.2% during the COVID-19 pandemic. About 6.3% of the study participants slept badly before the COVID-19 pandemic and the effects of sleeping badly and restlessly increased to 25.9% during the COVID-19 pandemic (<0.001). Of the participants, 19.3% participants were rated energy level before coronavirus pandemic as energized and decreased to 18.4% during COVID-19 pandemic with no significant change. However, a significant change was noticed in the energy level before the COVID-19 pandemic as lazy (5.7%) and increased to (18.4%) during COVID-19 (0.035) **(Table 7).**

**Table 7:** Sleep status of the respondents during COVID-19 pandemic.

Variables	Pre-COVID-19 n (%)	During-COVID-19 n (%)	p-Value (2-Sided)
**Hours of sleep per night**			
<7 hours	125 (35.9)	122 (35.1)	<0.001
7–9 hours	144 (41.4)	117 (33.6)	<0.001
>9 hours	79 (22.7)	109 (31.3)	<0.001
**How would you rate your sleep quality?**			
Very good	152 (43.7)	77 (22.1)	<0.001
Good	167 (48.0)	173 (49.7)	<0.001
Poor	29 (8.3)	98 (28.2)	<0.001
**Did you experience any of the following?**			
Slept badly and restlessly	22 (6.3)	90 (25.9)	<0.001
Hard to go to sleep	43 (12.4)	47 (13.5)	0.66
Woken up too early and not been able to get back to sleep	44 (12.6)	56 (16.1)	0.74
Woken up several times and found it difficult to get back to sleep	59 (17.0)	56 (16.4)	0,23
None	180 (51.7)	98 (28.2)	0.033
**Describe your energy level**			
Energized	67 (19.3)	64 (18.4)	0.22
Neutral	261 (75)	220 (63.2)	0.82
Lazy	20 (5.7)	64 (18.4)	0.035

## 4. Discussion

This community-based cross-sectional study assessed the lifestyle changes during COVID 19 pandemic in Gondar town, North West, Ethiopia between June and August 2021. Change in dietary habit due to COVID-19 is one of the outlines that show variations in the direction of good health habits with increased consumption of vegetables, fruits, legumes, and fish, but in other cases, they show changes toward higher consumption of sugary beverages which can inhibit the good habits and predispose for unhealthier habits [38,39]. The current study focused on meal consumption revealed that there are significant downgrade alterations of non-homemade food conceptions. This could be due to the fear of COVID-19 transmission from either restaurant delivery persons, or hygiene practices. This finding is in agreement with previous studies conducted in United Arab Emirates [30], Kuwait [40], and Saudi Arabia [41]. However, there is an increment in the frequency of food consumption ≥5 meals by 2% (0.01) during the COVID-19 pandemic. This finding is inconsistent with a study conducted in the United Arab Emirates [30].

In this study, the consumption of fruit, vegetable, milk and milk products, beard/rice/pasta, and sweet beverages was lower than the study conducted in the United Arab Emirates [30]. The variation in sweet drinks and another feeding style may be a change in lifestyle habits and economical status. The rate of food intake in the current study is also lower than the study conducted in Italy and China [28,42]. The difference in dietary habits may be due to changes compatible with greater adherence to the usual diet. These patterns indicate unhealthy eating behaviors during the period of the pandemic. This is constituent with previous similar studies reporting a transformation of the diet from a traditional diet to a more Westernized diet which is high in salt, cholesterol, energy, refined carbohydrates, and saturated fat, and low in fiber, vegetables, fruits, and polyunsaturated fats [43–46].

Performing regular physical activity is one of the mechanisms to reduce the effects of COVID-19 severity [39,47]. The current study revealed that about 46% of the respondents reported never engaging in any physical activity before the coronavirus pandemic, furthermore the percentage decreased to 29.9% during the pandemic (p = 0.002). The possible reason for the decrement of physical activity in the current study may be due to staying at home, improper understanding of the advantages of physical activity to general health, and lack of commitment to perform physical activity. This finding is consistent with a study conducted in Spain that a lower rate of people was engaged in physical activity [48]. However, the rate of physical activity was lower when compared to studies conducted in Sweden [49], Italy [28], and the University of Sarajevo [50]. The current study highlights the level of stress explained by physical, emotional, irritability, and tension as it predisposes poor health outcomes and leads to aggravating COVID-19 infection poor sleep. COVID-19 pandemic had a dramatic influence on lifestyle behaviors worldwide, including reduced engagement in physical activity and sports in general. Therefore, awareness concerning the importance of regular physical activity and its benefits on overall health is essential during such times [51,52].

During the COVID-19 pandemic higher levels of stress, anxiety, and depression have been detected among individuals [53–55]. The stress and irritabilities are poor indicators of health outcome and the current study revealed that about 11.8% and 12.4% of the study participants were physically exhausted in all parts of the time before and during the COVID-19 pandemic, respectively while 8.3% and 10.6% of the respondents emotionally exhausted before and during COVID-19 pandemic all part of the time. However, the level of irritability before the COVID -19 pandemic increased from 7.2% to 21.3% during the COCVID-19 pandemic. The respondents also exhibited increment of tension in large from 4.9% to 22.7% before and during the COVID-19 pandemic, respectively. The magnitude of the current study was higher when compared with the study conducted in Ethiopia with the magnitude of stress (11.1%) [56]. This higher magnitude may be due to prolonged stays at home and isolation despite applying COVID -19 prevention. However, the study finding is lower when compared with the study conducted in the United Kingdom [57], and Russia [58], but the level of physical exhaustion, emotional exhaustion, and irritability is in line with a study conducted in the United Arab Emirates, that before vs after the pandemic (13.3% vs 7.7% for physical exhaustion; 14.1% vs. 6.3% for emotional exhaustion; 13.5% vs. 6.9% for irritability; and 17.8% vs. 6.3% for tension), respectively [30].

Anxiety and stress could disrupt sleep quality during the night and energy levels during the day and it is clear that the quality of sleep affects the general health of the respondents [30]. In this study, 8.3% of the respondents had poor sleep quality pre-COVID-19, however, the report of poor quality of sleep was increased by 19.9% during the COVID-19 pandemic. The insomnia level of respondents was declared when the sleep habits < 7hrs and this finding is lower when compared with a similar study conducted in Ethiopia with a total rate of 71% [23]. This significant difference may be due to fear of the effects of the COVID-19 pandemic and habits of daytime sleep other than nighttime sleep. Regarding the quality of sleep, the rate of poor sleep quality was lower when compared with previous similar studies (39.5%) [59], and (35.2%) [60]. However, the level of sleep quality was higher than the study conducted in Russia of which 12.4% suffered from chronic sleep deprivation and about 50.2% of the study participants did not get the required amount of sleep due to a high workload [58]. This difference may be due to fear of loneliness in the case of corona and fear of separation from family members as well as fear of the diseases.

## 5. Limitation of the study

Since it was a cross-sectional study design, it did not display the conditions of cause and effect association. In addition, the study was limited to the Gondar town, which may not be representative of the rural area.

## 6. Conclusion

The promotion of correct lifestyles is crucial for the protection of health, but it becomes even more so in case of forced confinement at home. The current study demonstrates that there is a noticeable alteration in food consumption, food choices, regular mealtime, mental exhaustion, and practice of physical activity. Sleeping habits, level of restlessness, and insomnia also changed during COVID -19.

## 7. Recommendations

As there is poor practice of physical activity, the study participants should be moved toward home-based physical activity programs. Psychological changes may compromise the health status of the individual therefore, psychological counseling and positive reassurance are necessary. Adequate sleep is crucial, hence avoiding frustration, eating adequate and appropriate food, avoiding caffeine intake, and Coca-Cola beverage intakes are recommended to promote good sleep quality. Even though quarantine is an indispensable measure to control the transmission of the virus and protect public health, these findings should be taken into consideration for future regulations in Ethiopia.

## Supporting information

S1 File This is a questionnaire. (DOCX)

S2 File This is a questionnaire. (DOCX)

S3 File This is SPSS data. (SAV)
