# Supplementary material for: Assessment of lifestyle changes during coronavirus disease 2019 pandemic in Gondar town, Northwest Ethiopia
Source: PLoS One. 2022 Mar 18;17(3):e0264617. doi: 10.1371/journal.pone.0264617 (PMC8932614; doi:10.1371/journal.pone.0264617)
Supplement: S1 File — (DOCX) [file pone.0264617.s001.docx]

# 10. ANNEX

**10.1. Consent to participate for this study**

Dear sir/madam I am______________________________________. I am working with a research team to assess Assessment of life style changes during COVID 19 pandemic in north Gondar town, Ethiopia. The information you provide will be a very important input in the understanding of the topic at hand and devise and implement interventions for any identified problems. It will take only 15-25 minutes. You have the right not to answer any question that you don’t want to respond to. You can also quit at any time if you want to do so. If you have any question about this study for further clarification, you can ask.

So, are you willing to participate in this study?

Yes No

If yes, start the data collection. Respondent’s no. ___________ Date ______________________

**Please read each question and encircle your choice or fill in the space provided**

Table 8: Socio-demographic characteristics and other background information

| **No.** | **Question/variable** | **Options/ answer** |
| --- | --- | --- |
| 1 | Which age group do you belong to? | 1. 18-25 2. 26-35 3. 36-45 4. 46-55 5. >56 |
| 2 | What is your gender? | 1. Female 2. Male |
| 3 | What is your religion? | 1. Orthodox 2. Muslim 3. Protestant 4. Other (specify)_____________ |
| 4 | What is your marital status? | 1. Married 2. Single 3. Widowed 4. Divorced |
| 5 | How many kids do you have? | 1. None 2. 1–2 3. ≥ 3 |
| 6 | What is your education Level? | 1. Illiterate 2. Less than high school 3. High school 4. College/Diploma 5. Bachelor’s degree 6. Higher than bachelor’s degree |
| 7 | What is your employment status? | 1. Full-time 2. Part-time 3. Self-employed 4. Student 5. Unemployed 6. Retired |
| 8 | Amount of money you earn per month by any means | 1. < 2500 birr 2. 2500-5000 birr 3. >5000 birr |
| 9 | Did your weight change during coronavirus pandemic? | 1. Lost weight 2. Gained weight 3. Maintained weight 4. I don’t know 5. Do not know |
| 10 | What option describes your general state of health during Covid-19? | 1. Excellent 2. Very good 3. Good 4. Fair 5. Poor |

1. **Sources of information**

| **No.** | **Question/variable** | **Options/ answer** |
| --- | --- | --- |
| 1 | Where do you get health-related information from? (choose all that apply) | 1. Local and international health authorities 2. Websites and social media 3. Healthcare professionals 4. Television 5. Newspapers 6. Friends and family |
| 2 | Where do you get food and nutrition related information from? (choose all that apply) | 1. Local and international health authorities 2. Websites and social media 3. Healthcare professionals 4. Television 5. Newspapers   Friends and family |

1. **Eating Habits**

| **No.** | **Question/variable** | **Options/ answer** |
| --- | --- | --- |
| 1 | Before coronavirus pandemic, most of your consumed meals during the week were? | 1. Homemade 2. None-homemade |
| 2 | During coronavirus pandemic most of your consumed meals during the week are? | 1. Homemade 2. None-homemade |
| 3 | How many meals did you eat per day before coronavirus pandemic? | 1. 1-2 2. 3-4 3. More than 5 |
| 4 | How many meals do you eat per day during coronavirus pandemic? | 1. 1-2 2. 3-4 3. More than 5 |
| 5 | Did you use to eat breakfast on most days of the week before coronavirus pandemic? | 1. Yes 2. No |
| 6 | During coronavirus pandemic, do you eat breakfast on most days of the week? | 1. Yes 2. No |
| 7 | Before coronavirus pandemic, did you use to skip meals? | 1. Yes 2. No |
| 8 | If yes to the above question, why is that? | 1. To reduce food intake 2. Lack of time 3. To lose weight 4. Lack of appetite 5. Fasting |
| 9 | During coronavirus pandemic, are you skipping meals? | 1. Yes 2. No |
| 10 | If yes to the above question, why is that? | 1. To reduce food intake 2. Lack of time 3. To lose weight 4. Lack of appetite 5. Fasting |
| 11 | Before coronavirus pandemic, how much water did you use to drink daily? | 1. 1-4 cups 2. 5-7 cups 3. 8 cups or more |
| 12 | During coronavirus pandemic, how much water do you drink daily? | 1. 1-4 cups 2. 5-7 cups 3. 8 cups or more |

13. How often do you eat the following foods listed below?

| Food Item | Never | 1-4 times/week | Once/day | 2-3 times/day | 4 or more times/day |
| --- | --- | --- | --- | --- | --- |
| Fruits |  |  |  |  |  |
| Vegetables |  |  |  |  |  |
| Milk and milk products |  |  |  |  |  |
| Meat/Chicken/Fish |  |  |  |  |  |
| Bread/rice/pasta |  |  |  |  |  |
| Sweets/ desserts |  |  |  |  |  |
| Coffee/Tea |  |  |  |  |  |
| Sweet drinks (soft drinks, canned juice, etc. |  |  |  |  |  |

1. **Shopping**

| **No.** | **Question/variable** | **Options/ answer** |
| --- | --- | --- |
| 1 | Did you usually prepare a list before grocery shopping before coronavirus pandemic? | 1. Yes 2. No |
| 2 | During coronavirus pandemic, do you usually prepare a list before grocery shopping? | 1. Yes 2. No |
| 3 | Did you start stocking up on food before coronavirus pandemic? | 1. Yes 2. No |
| 4 | During coronavirus pandemic, do you start stocking up on food? | 1. Yes 2. No |
| 5 | Would you rather order your groceries online (delivered to your house) before coronavirus pandemic? | 1. Yes 2. No |
| 6 | During coronavirus pandemic, would you rather order your groceries online (delivered to your house) | 1. Yes 2. No |
| 7 | Did you check food labels before purchasing before coronavirus pandemic? | 1. Yes 2. No |
| 8 | During coronavirus pandemic, do you check food labels before purchasing? | 1. Yes 2. No |
| 9 | Did you clean and sanitize your groceries before storage before coronavirus pandemic? | 1. Yes 2. No |
| 10 | During coronavirus pandemic, do you clean and sanitize your groceries before storage | 1. Yes 2. No |

1. **Physical Activity**

| **No.** | **Question/variable** | **Options/ answer** |
| --- | --- | --- |
| 1 | Did you use to exercise before coronavirus pandemic? | 1. Never 2. 1-3 times/week 3. >3 times/week |
| 2 | During coronavirus pandemic, do you do any exercise (walking, running, equipment, etc.)? | 1. Never (skip to Question 3) 2. 1-3 times/week 3. >3 times/week |
| 3 | Before coronavirus pandemic, did you use to do household chores? | 1. Never 2. 1-3 times/week 3. 4-5 times/week 4. Everyday |
| 4 | During coronavirus pandemic, do you do household chores? | 1. Never 2. 1-3 times/week 3. 4-5 times/week 4. Everyday |
| 5 | Before coronavirus pandemic, how much time did you spend on the computer for work/ study daily? | 1. None 2. 1-2 hours 3. 3-5 hours 4. More than 5 hours |
| 6 | During coronavirus pandemic, how much time do you spend on the computer for work/ study daily? | 1. None 2. 1-2 hours 3. 3-5 hours 4. More than 5 hours |
| 7 | Before coronavirus pandemic, how much time did you spend daily on Television, computer, social media for entertainment? | 1. Less than 30 minutes 2. 1-2 hours 3. 3-5 hours 4. More than 5 hours |
| 8 | During coronavirus pandemic, how much time do you spend daily on Television, computer, social media for entertainment? | 1. Less than 30 minutes 2. 1-2 hours 3. 3-5 hours 4. More than 5 hours |

1. **Stress and Irritability**
2. How often have you experienced the following before coronavirus pandemic?

|  | All the time | A large part of the time | Part of the time | A small part of the time | Not at all |
| --- | --- | --- | --- | --- | --- |
| Physically exhausted |  |  |  |  |  |
| Emotionally exhausted |  |  |  |  |  |
| Irritable |  |  |  |  |  |
| Tense |  |  |  |  |  |

1. How often have you experienced the following during coronavirus pandemic?

|  | All the time | A large part of the time | Part of the time | A small part of the time | Not at all |
| --- | --- | --- | --- | --- | --- |
| Physically exhausted |  |  |  |  |  |
| Emotionally exhausted |  |  |  |  |  |
| Irritable |  |  |  |  |  |
| Tense |  |  |  |  |  |

1. **Sleep**

| **No.** | **Question/variable** | **Options/ answer** |
| --- | --- | --- |
| 1 | Before coronavirus pandemic, how many hours did you sleep per night? | 1. Less than 7 hours 2. 7-9 hours 3. More than 9 hours |
| 2 | During coronavirus pandemic, how many hours are you sleeping per night? | 1. Less than 7 hours 2. 7-9 hours 3. More than 9 hours |
| 3 | How would you rate your sleep quality before coronavirus pandemic? | 1. Very good 2. Good 3. Poor |
| 4 | How would you rate your sleep quality during coronavirus pandemic? | 1. Very good 2. Good 3. Poor |
| 5 | Before coronavirus pandemic, did you experience any of the following? (choose all that apply) | 1. Slept badly and restlessly 2. Hard to go to sleep 3. Woken up too early and not been able to get back to sleep 4. Woken up several times and found it difficult to get back to sleep 5. None of the above |
| 6 | During coronavirus pandemic, do you experience any of the following? (choose all that apply) | 1. Slept badly and restlessly 2. Hard to go to sleep 3. Woken up too early and not been able to get back to sleep 4. Woken up several times and found it difficult to get back to sleep 5. None of the above |
| 7 | How would you describe your energy level before coronavirus pandemic? | 1. Energized 2. Neutral 3. Lazy |
| 8 | How would you describe your energy level during coronavirus pandemic? | 1. Energized 2. Neutral 3. Lazy |
