# Supplementary material for: Assessment of lifestyle changes during coronavirus disease 2019 pandemic in Gondar town, Northwest Ethiopia
Source: PLoS One. 2022 Mar 18;17(3):e0264617. doi: 10.1371/journal.pone.0264617 (PMC8932614; doi:10.1371/journal.pone.0264617)
Supplement: S2 File — (DOCX) [file pone.0264617.s002.docx]

**ለዚህ ጥናት ለመሳተፍ ስምምነት**

የተከበሩ አቶ / ወሮ እኔ____________________________________. በሰሜን ጎንደር ከተማ በኢትዮጵያ በተከሰተ ወረርሽኝ COVID 19 በተከሰተበት ወቅት የአኗኗር ዘይቤ ለውጦች ግምገማ ለመገምገም ከአንድ የምርምር ቡድን ጋር እየሰራሁ ነው ፡፡ እርስዎ ያቀረቡት መረጃ በእጃቸው ላይ ያለውን ርዕስ ለመረዳት በጣም አስፈላጊ ግብዓት ይሆናል እና ለተለዩ ችግሮች ጣልቃ ገብነትን ለመንደፍ እና ተግባራዊ ለማድረግ ነው ፡፡ የሚወስደው ከ15-25 ደቂቃዎች ብቻ ነው ፡፡ እርስዎ መመለስ የማይፈልጉትን ማንኛውንም ጥያቄ ላለመመለስ መብት አለዎት። እንዲሁም ይህን ማድረግ ከፈለጉ በማንኛውም ጊዜ ማቆም ይችላሉ። ለተጨማሪ ማብራሪያ ስለዚህ ጥናት ማንኛውም ጥያቄ ካለዎት መጠየቅ ይችላሉ ፡፡

ስለዚህ ፣ በዚህ ጥናት ውስጥ ለመሳተፍ ፈቃደኛ ነዎት?

አዎ አይ

አዎ የእርስዎ መልስ ከሆነ፣ የመረጃ ማሰባሰቡን ይጀምሩ ተጠሪ ቁ. ___________ ቀን ______________________

እባክዎ እያንዳንዱን ጥያቄ ያንብቡ እና የመረጡትን ይክፈሉ ወይም የተሰጠውን ቦታ ይሙሉ

I. ማህበራዊ-ስነ-ህዝብ ባህሪዎች እና ሌሎች የጀርባ መረጃ

| **No.** | **ጥያቄ / ተለዋዋጭ** | **አማራጮች / መልስ** |
| --- | --- | --- |
| 1 | ከየትኛው የዕድሜ ክልል ውስጥ ነዎት? | ሀ. 18-25  ለ. 26-35  ሐ. 36-45  መ. 46-55  ሠ. > 56 |
| 2 | የእርስዎ ፆታ ምንድነው? | ሀ. ሴት  ለ. ወንድ |
| 3 | ሃይማኖትዎ ምንድን ነው? | ሀ. ኦርቶዶክስ  ለ. ሙስሊም  ሐ. ፕሮቴስታንት  መ. ሌላ (ይግለጹ |
| 4 | የጋብቻ ሁኔታዎ ምንድ ነው? | ሀ. ያገባ  ለ. ያላገባ  ሐ. መበለት  መ. የተፋታች |
| 5 | ስንት ልጆች አሉዎት? | ሀ. የለም  ለ. 1-2  ሐ. ≥ 3 |
| 6 | የትምህርት ደረጃዎ ምንድ ነው? | ሀ. ማንበብና መጻፍ  ለ. ከሁለተኛ ደረጃ ትምህርት ቤት ያነሰ  ሐ. ሁለተኛ ደረጃ ትምህርት ቤት  መ. ኮሌጅ / ዲፕሎማ  ሠ. የመጀመሪያ ዲግሪ  ረ. ከባችለር ዲግሪ ከፍ ያለ |
| 7 | የቅጥር ሁኔታዎ ምንድ ነው? | ሀ. ሙሉ ግዜ  ለ. የትርፍ ጊዜ  ሐ. በግል ተዳዳሪ  መ. ተማሪ  ሠ. ሥራ አጥነት  ረ. ጡረታ ወጥቷል |
| 8 | በማንኛውም መንገድ በወር የሚያገኙት የገንዘብ መጠን | ሀ. <2500 ብር  ለ. 2500-5000 ብር  ሐ. > 5000 ብር |
| 9 | በኮሮናቫይረስ ወረርሽኝ ወቅት ክብደትዎ ተለውጧል? | ሀ. ክብደት ቀንሷል  ለ. ክብደት አግኝቷል  ሐ. ክብደትን ጠብቆ መቆየት  መ. እኔ አላውቅም |
| 10 | በኮቪ -19 ወቅት አጠቃላይ የጤና ሁኔታዎን የሚገልጽ ምን አማራጭ አለ? | ሀ. በጣም ጥሩ  ለ. ጥሩ  ሐ. መካከለኛ  መ. ደካማ |
|  |  |  |

1. **የመረጃ ምንጮች**

| **No.** | **ጥያቄ / ተለዋዋጭ** | **አማራጮች / መልስ** |
| --- | --- | --- |
| 1 | ከጤና ጋር የተዛመደ መረጃ ከየት ነው የሚያገኙት? (የሚመለከተውን ሁሉ ይምረጡ) | ሀ. የአከባቢ እና ዓለም አቀፍ የጤና ባለሥልጣናት  ለ. ድርጣቢያዎች እና ማህበራዊ አውታረ መረቦች  ሐ. የጤና እንክብካቤ ባለሙያዎች  መ. ቴሌቪዥን  ሠ. ጋዜጣዎች  ረ. ጓደኞች እና ቤተሰቦች |
| 2 | ምግብ እና የተመጣጠነ ምግብ ነክ መረጃዎችን ከየት ያመጣሉ? (የሚመለከተውን ሁሉ ይምረጡ) | ሀ. የአከባቢ እና ዓለም አቀፍ የጤና ባለሥልጣናት  ለ. ድርጣቢያዎች እና ማህበራዊ አውታረ መረቦች  ሐ. የጤና እንክብካቤ ባለሙያዎች  መ. ቴሌቪዥን  ሠ. ጋዜጣዎች  ጓደኞች እና ቤተሰቦች |

**አማራጮች / መልስ**

**አማራጮች / መልስ**

**አማራጮች / መልስ**

**አማራጮች / መልስ**

1. **የአመጋገብ ልማድ**

| **No.** | **ጥያቄ / ተለዋዋጭ** | **አማራጮች / መልስ** |
| --- | --- | --- |
| 1 | ከኮሮናቫይረስ ወረርሽኝ በፊት በሳምንቱ ውስጥ አብዛኛዎቹ የሚበሉት ምግቦች የት ያገኛሉ? | ሀ. በቤት ውስጥ የተሰራ  ለ. ከ ቤት ውጭ የተሰራ |
| 2 | በኮሮናቫይረስ ወረርሽኝ ወቅት በሳምንቱ ውስጥ አብዛኛዎቹ የሚበሉት ምግቦች የት ያገኛሉ? | ሀ. በቤት ውስጥ የተሰራ  ለ. ከ ቤት ውጭ የተሰራ |
| 3 | ከኮሮናቫይረስ ወረርሽኝ በፊት በየቀኑ ስንት ጊዜ ምግብ ይመገቡ ነበር? | ሀ. 1-2  ለ. 3-4  ሐ. ከ 5 በላይ |
| 4 | በኮሮናቫይረስ ወረርሽኝ ወቅት በቀን ስንት ጊዜ ምግብ ይመገባሉ? | ሀ. 1-2  ለ. 3-4  ሐ. ከ 5 በላይ |
| 5 | በኮሮናቫይረስ ወረርሽኝ ከመከሰቱ በፊት በሳምንቱ ቀናት ውስጥ አብዛኛውን ጊዜ ቁርስ የመብላት ልምድ አለዎት ወ? | ሀ. አዎ  ለ. አይ |
| 6 | በኮሮናቫይረስ ወረርሽኝ ወቅት በሳምንቱ ብዙ ቀናት ቁርስ ይመገባሉ? | ሀ. አዎ  ለ. አይ |
| 7 | ከኮሮናቫይረስ ወረርሽኝ በፊት ምግብን ለመዝለል ይጠቀሙ ነበር? | ሀ. አዎ  ለ. አይ |
| 8 | ከላይ ለተጠቀሰው ጥያቄ አዎ ከሆነ ለምን እንዲህ ሆነ? | ሀ. የምግብ ቅበላን ለመቀነስ  ለ. የጊዜ እጥረት  ሐ. ክብደት ለመቀነስ  መ. የምግብ ፍላጎት እጥረት  ሠ. ጾም |
| 9 | በኮሮናቫይረስ ወረርሽኝ ወቅት ምግብ ሰአት ያሳልፋሉ? | ሀ. አዎ  ለ. አይ |
| 10 | ከላይ ለተጠቀሰው ጥያቄ አዎ ከሆነ ለምን እንዲህ ሆነ? | ሀ. የምግብ ቅበላን ለመቀነስ  ለ. የጊዜ እጥረት  ሐ. ክብደት ለመቀነስ  መ. የምግብ ፍላጎት እጥረት  ሠ. ጾም |
| 11 | ከኮሮናቫይረስ ወረርሽኝ በፊት በየቀኑ ምን ያህል ውሃ ይጠጡ ነበር? | ሀ. 1-4 ኩባያዎች  ለ. 5-7 ኩባያዎች  ሐ. 8 ኩባያዎች ወይም ከዚያ በላይ |
| 12 | በኮሮናቫይረስ ወረርሽኝ ወቅት በየቀኑ ምን ያህል ውሃ ይጠጣሉ? | ሀ. 1-4 ኩባያዎች  ለ. 5-7 ኩባያዎች  ሐ. 8 ኩባያዎች ወይም ከዚያ በላይ |

13. ከዚህ በታች የተዘረዘሩትን የሚከተሉትን ምግቦች ምን ያህል ጊዜ ይመገባሉ?

| የምግብ እቃ | በጭራሽ | 1-4 ጊዜ / ሳምንት | በቀን አንዴ | በቀን 2-3 ጊዜ | በቀን 4 ወይም ከዚያ በላይ |
| --- | --- | --- | --- | --- | --- |
| ፍራፍሬዎች |  |  |  |  |  |
| አትክልቶች |  |  |  |  |  |
| የወተት እና የወተት ተዋጽኦዎች |  |  |  |  |  |
| ስጋ / ዶሮ / ዓሳ |  |  |  |  |  |
| ዳቦ / ሩዝ / ፓስታ |  |  |  |  |  |
| ጣፋጮች / ማጣጣሚያ ነገሮች |  |  |  |  |  |
| ቡና / ሻይ |  |  |  |  |  |
| ጣፋጭ መጠጦች (ለስላሳ መጠጦች ፣ የታሸገ ጭማቂ ፣ ወዘተ) ፡፡ |  |  |  |  |  |

1. **ግብይት**

| **No.** | **ጥያቄ / ተለዋዋጭ** | **አማራጮች / መልስ** |
| --- | --- | --- |
| 1 | አብዛኛውን ጊዜ የኮሮናቫይረስ ወረርሽኝ ከመከሰቱ በፊት ዕቃ ለመግዛት ዝርዝር አዘጋጅተው ይሄዱ ነበር ወይ? | ሀ. አዎ ለ. አይ |
| 2 | የኮሮናቫይረስ ወረርሽኝ ወቅት አብዛኛውን ጊዜ ዕቃ ከመግዛትህ በፊት ዝርዝር ታዘጋጃለህ/ሽ? | ሀ. አዎ  ለ. አይ |
| 3 | የኮሮናቫይረስ ወረርሽኝ ከመከሰቱ በፊት ምግብ ማከማቸት አለዎት ? | ሀ. አዎ  ለ. አይ |
| 4 | የኮሮናቫይረስ ወረርሽኝ በሚከሰትበት ወቅት ምግብ ማከማቸት ጀምረሃል/ሽ? | ሀ. አዎ  ለ. አይ |
| 5 | የኮሮናቫይረስ ወረርሽኝ ከመከሰቱ በፊት ሸቀጣ ሸቀጦችን በኢንተርኔት (ወደ ቤትህ ማድረስ) ማዘዝ ትመርጣለህ? | ሀ. አዎ  ለ. አይ |
| 6 | የኮሮናቫይረስ ወረርሽኝ በሚከሰትበት ጊዜ የምግብ ሸቀጦችህን በኢንተርኔት ማዘዝ ጅምርሃል/ሻል | ሀ. አዎ  ለ. አይ |
| 7 | ከኮሮናቫይረስ ወረርሽኝ በፊት ከመግዛትዎ በፊት የምግብ ንጥረነገሮችን ፈትሸዋል? | ሀ. አዎ  ለ. አይ |
| 8 | በኮሮናቫይረስ ወረርሽኝ ወቅት ከመግዛትዎ በፊት የምግብ ንጥረነገሮችን ይመረምራሉ? | ሀ. አዎ  ለ. አይ |
| 9 | ከኮሮናቫይረስ ወረርሽኝ በፊት የገዙትን እቃወች ለማስቀመጥ ማስቀመጫውን ያጸዳሉ ? | ሀ. አዎ  ለ. አይ |
| 10 | በኮሮናቫይረስ ወረርሽኝ ወቅት የምግብ ሸቀጣ ሸቀጦቹን ከማጠራቀምዎ በፊት ያፀዳሉ? | ሀ. አዎ  ለ. አይ |

1. **አካላዊ እንቅስቃሴ**

| **No.** | **ጥያቄ / ተለዋዋጭ** | **አማራጮች / መልስ** |
| --- | --- | --- |
| 1 | ከኮሮናቫይረስ ወረርሽኝ በፊት የአካል ብቃት እንቅስቃሴን ይጠቀሙ ነበር? | ሀ. በጭራሽ  ለ. 1-3 ጊዜ / ሳምንት  ሐ. > በሳምንት 3 ጊዜ |
| 2 | በኮሮናቫይረስ ወረርሽኝ ወቅት ማንኛውንም የአካል ብቃት እንቅስቃሴ ያካሂዳሉ (በእግር ፣ በሩጫ ፣ በመሣሪያ ፣ ወዘተ)? | ሀ. በጭራሽ (ወደ ጥያቄ 3 ዝለል)  ለ. 1-3 ጊዜ / ሳምንት  ሐ. > በሳምንት 3 ጊዜ |
| 3 | ከኮሮናቫይረስ ወረርሽኝ በፊት የቤት ውስጥ ሥራዎችን ይሠሩ ነበር? | ሀ. በጭራሽ  ለ. 1-3 ጊዜ / ሳምንት  ሐ. በሳምንት 4-5 ጊዜ  መ. በየቀኑ |
| 4 | በኮሮናቫይረስ ወረርሽኝ ወቅት የቤት ውስጥ ሥራዎችን ያከናውናሉ? | ሀ. በጭራሽ  ለ. 1-3 ጊዜ / ሳምንት  ሐ. በሳምንት 4-5 ጊዜ  መ. በየቀኑ |
| 5 | ከኮሮናቫይረስ ወረርሽኝ በፊት በኮምፒተር ውስጥ በየቀኑ ለስራ / ለማጥናት ምን ያህል ጊዜ አሳልፈዋል? | ሀ. በጭራሽ  ለ. 1-2 ሰዓታት  ሐ. ከ3-5 ሰዓታት  መ. ከ 5 ሰዓታት በላይ |
| 6 | በኮሮናቫይረስ ወረርሽኝ ወቅት በየቀኑ ለሥራ / ለማጥናት በኮምፒዩተር ላይ ምን ያህል ጊዜ ያጠፋሉ? | ሀ. በጭራሽ  ለ. 1-2 ሰዓታት  ሐ. ከ3-5 ሰዓታት  መ. ከ 5 ሰዓታት በላይ |
| 7 | ከኮሮናቫይረስ ወረርሽኝ በፊት በቴሌቪዥን ፣ በኮምፒተር ፣ በማህበራዊ ሚዲያ ለመዝናኛ በየቀኑ ምን ያህል ጊዜ ያጠፋሉ? | ሀ. ከ 30 ደቂቃዎች በታች  ለ. 1-2 ሰዓታት  ሐ. ከ3-5 ሰዓታት  መ. ከ 5 ሰዓታት በላይ |
| 8 | በኮሮናቫይረስ ወረርሽኝ ወቅት ለመዝናኛ በየቀኑ በቴሌቪዥን ፣ በኮምፒተር ፣ በማህበራዊ ሚዲያ ምን ያህል ጊዜ ያጠፋሉ? | ሀ. ከ 30 ደቂቃዎች በታች  ለ. 1-2 ሰዓታት  ሐ. ከ3-5 ሰዓታት  መ. ከ 5 ሰዓታት በላይ |

1. ውጥረት እና ብስጭት
2. 2. ከኮሮናቫይረስ ወረርሽኝ በፊት የሚከተሉትን ስንት ጊዜ አጋጥመዎታል?

|  | **ሁልጊዜ** | **የጊዜ ትልቅ ክፍል** | **የጊዜ ክፍል** | **የጊዜ ትንሽ ክፍል** | **በፍፁም** |
| --- | --- | --- | --- | --- | --- |
| **አካላዊ ድካም** |  |  |  |  |  |
| **አዕምሮ ድካም** |  |  |  |  |  |
| **መረበሽ** |  |  |  |  |  |
| **ጭንቀት** |  |  |  |  |  |

1. **በኮሮናቫይረስ ወረርሽኝ ወቅት የሚከተሉትን ስንት ጊዜ አጋጥመዎታል?**

|  | **ሁልጊዜ** | **የጊዜ ትልቅ ክፍል** | **የጊዜ ክፍል** | **የጊዜ ትንሽ ክፍል** | **በፍፁም** |
| --- | --- | --- | --- | --- | --- |
| **አካላዊ ድካም** |  |  |  |  |  |
| **አዕምሮ ድካም** |  |  |  |  |  |
| **መረበሽ** |  |  |  |  |  |
| **ጭንቀት** |  |  |  |  |  |

1. **እንቅልፍ**

| **No.** | **ጥያቄ / ተለዋዋጭ** | **አማራጮች / መልስ** |
| --- | --- | --- |
| 1 | የኮሮናቫይረስ ወረርሽኝ ከመከሰቱ በፊት በእያንዳንዱ ሌሊት ምን ያህል ሰዓት ይተኙ ነበር? | ሀ. ከ 7 ሰዓታት በታች  ለ. 7-9 ሰዓታት  ሐ. ከ 9 ሰዓታት በላይ |
| 2 | የኮሮናቫይረስ ወረርሽኝ በሚከሰትበት ጊዜ በእያንዳንዱ ሌሊት ምን ያህል ሰዓት ትተኛለህ/ሽ? | ሀ. ከ 7 ሰዓታት በታች  ለ. 7-9 ሰዓታት  ሐ. ከ 9 ሰዓታት በላይ |
| 3 | ከኮሮናቫይረስ ወረርሽኝ በፊት የእንቅልፍ ጥራትዎን እንዴት ይመዘኑታል? | ሀ. በጣም ጥሩ  ለ. ጥሩ  ሐ. ደካማ |
| 4 | በኮሮናቫይረስ ወረርሽኝ ወቅት የእንቅልፍዎን ጥራት እንዴት ይመዘኑታል? | ሀ. በጣም ጥሩ  ለ. ጥሩ  ሐ. ደካማ |
| 5 | የኮሮቫይረስ ወረርሽኝ ከመከሰቱ በፊት ከሚከተሉት ውስጥ አንዳቸውም አጋጥመውዎታል? (የሚመለከተውን ሁሉ ይምረጡ) | ሀ. በመጥፎ እና ያለ እረፍት መተኛት  ለ. እንቅልፍ ለመተኛት መቸገር  ሐ. ቶሎ ተነስቶ ወደ እንቅልፍ መተኛት ግን እንቅልፍ አለምመጣት መቸገር  መ. ብዙ ጊዜ ተነስቶ ወደ እንቅልፍ መተኛት ከባድ ሆኖበታል  ሠ. ከላይ ከተዘረዘሩት ውስጥ አንዳቸውም አይደሉም |
| 6 | በኮሮናቫይረስ ወረርሽኝ ወቅት ከሚከተሉት ውስጥ አንዱን ያጋጥሙዎታል? (የሚመለከተውን ሁሉ ይምረጡ) | ሀ. በመጥፎ እና ያለ እረፍት መተኛት ተቸግረዋል  ለ. ለመተኛት ከባድ  ሐ. ቶሎ ተነስቶ ወደ እንቅልፍ መተኛት ግን እንቅልፍ አለመመጣት መቸገር  መ. ብዙ ጊዜ ተነስቶ ወደ እንቅልፍ መተኛት ከባድ ሆኖበታል  ሠ. ከላይ ከተዘረዘሩት ውስጥ አንዳቸውም አይደሉም |
| 7 | ከኮሮናቫይረስ ወረርሽኝ በፊት የኃይልዎን ደረጃ እንዴት ይገልጹታል? | ሀ. ኃይል ነበረኝ  ለ. የተለመደ  ሐ. ሰነፍ |
| 8 | በኮሮናቫይረስ ወረርሽኝ ወቅት የኃይልዎን ደረጃ እንዴት ይገልፁታል? | ሀ. ኃይል ነበረኝ  ለ. የተለመደ  ሐ. ሰነፍ |
